# Cross-sectional analysis of triglycerides and serum uric acid association in ovarian cancer patients

**DOI:** 10.3389/fonc.2025.1713010

**Published:** 2026-01-15

**Authors:** Shuqi Zhang, Peiyao Li, Haifeng Xu, Xiaoli Zhao, Han Xiao, Wenjuan Li, Ping Li, Fang Chen, Lixia Zhu

**Affiliations:** 1 Department of Obstetrics and Gynecology, Gusu School, Nanjing Medical University, The First People’s Hospital of Kunshan, Suzhou, Jiangsu, China; 2 Department of Obstetrics and Gynecology, Affiliated Kunshan Hospital of Jiangsu University, Suzhou, Jiangsu, China; 3 Department of Radiotherapy and Oncology, Affiliated Kunshan Hospital of Jiangsu University, Suzhou, Jiangsu, China; 4 Department of Pathology, Affiliated Kunshan Hospital of Jiangsu University, Suzhou, Jiangsu, China

**Keywords:** body mass index, metabolism, ovarian cancer, serum uric acid, triglycerides

## Abstract

**Background:**

Although numerous studies have investigated the association between triglycerides (TG) and serum uric acid (SUA) in various populations, this association has not yet been explored in patients with ovarian cancer (OC). This article aims to examine the association between TG and SUA specifically in OC patients.

**Methods:**

This was a retrospective cross-sectional examination of data from 724 OC patients sourced from the Affiliated Kunshan Hospital of Jiangsu University database, hospitalized between December 2014 and May 2025. The baseline TG served as the exposure variable, whereas SUA levels constituted the study outcome. When analyzing this association, adjustments were made for age, various baseline clinical and laboratory parameters, followed by the fitting of separate univariate and multivariate linear regression models. This association was further characterized through smooth curve fitting, multiple regression equations, and threshold effect analyses.

**Results:**

A non-linear association between TG and SUA levels was discerned and modeled using a piecewise linear regression with an inflection point at 2.82 mmol/L. The analysis using piecewise multivariate linear methods indicated a substantial positive association between TG and SUA at TG levels below 2.82 mmol/L (β =52.73, 95% CI: 41.99 to 63.74, P < 0.01).

**Conclusions:**

This article reveals a non-linear association between TG and SUA, identifying a potential threshold effect. These findings offer new insights into their association in OC patients. However, given the cross-sectional design, these results represent associative rather than causal relationship and should be considered hypothesis-generating for future prospective studies.

## 1 Introduction

Ovarian cancer (OC) is one of the most common malignant tumors in women, with an increasing incidence that presents a considerable public health challenge worldwide (1). Upon diagnosis, patients face multiple obstacles that extend beyond physical pain and discomfort, encompassing considerable psychological and emotional distress (2). During the treatment process, various factors critically influence a patient’s physiological state, including individual nutritional status, fluctuations in hormone levels, the presence of chronic conditions, and the physical changes induced by therapeutic interventions (3). For instance, treatment modalities such as chemotherapy and radiotherapy often lead to substantial nutritional deficiencies and weight changes, which directly impact physiological functions and immune competence (4).

Serum uric acid (SUA) is the final product of purine metabolism, and hyperuricemia develops either due to excessive production or reduced excretion of SUA. When purine metabolism is disrupted and SUA production is elevated, or when factors such as renal tubule dysfunction or gut microbiota imbalance leading to decreased SUA excretion, SUA tends to accumulate in the bloodstream, causing elevated SUA levels. Additionally, impaired urinary excretion of SUA further contributes to the development of hyperuricemia (5, 6). When SUA levels surpass their solubility limit, SUA precipitates as urate crystals, which can trigger acute, painful attacks of arthritis and lead to the development of gout (6).

Triglycerides (TG) are neutral lipids consisting of three fatty acids esterified to a glycerol backbone, and are stored as lipid droplets within adipocytes (7). Moreover, TG are a type of fat that serves as a major source of energy for the human body. Chemically, they consist of three fatty acid chains attached to a glycerol molecule. TG are primarily stored in fat cells (adipocytes) and are released into the bloodstream to meet energy needs between meals. High levels of TG in the blood may increase the risk of cardiovascular diseases (8).

Research indicates that OC patients not only experience an elevated risk of reduced survival rates but also confront a range of psychological health issues, especially among long-term survivors (9). Key psychological challenges include anxiety, depression, and concerns regarding future survival, all of which significantly diminish overall quality of life (10). Existing literature substantiates that abnormalities in TG and SUA levels are closely linked to prognosis in various cancers and the risk of cardiovascular diseases (11). Current research suggests that TG are associated with hyperuricemia in obese individuals, but there is limited evidence of their association with OC patients (12). Elevated levels of TG and SUA serve as critical biomarkers of potential metabolic dysfunction, underscoring the importance for healthcare providers to closely monitor patients’ physiological well-being (13). Furthermore, these metabolic indicators may interact with the psychological challenges faced by patients, subsequently influencing their overall health status (14).

Investigating the correlation between TG and SUA levels in OC patients is of paramount importance (15). Preliminary studies have established a correlation between SUA and TG levels in adult populations, a finding that has been corroborated by multiple investigations (11). For instance, Tan et al. found significant association between SUA and TG levels across adult populations, highlighting the role of SUA as a marker for metabolic dysregulation (16). Additionally, Chu et al. have showed a substantial positive association between SUA and TG levels among Chinese children and adolescents with short stature hence strengthening the association between these parameters (17). However, research specifically focusing on the association between SUA and TG in the context of OC remains limited (11, 15). The aim of this study is to investigate the association between TG and SUA in OC patients, which can help guide treatment strategies and improve disease management (18).

## 2 Materials and methods

### 2.1 Study design and population

This study conducted a retrospective cross-sectional analysis, utilizing data gathered from December 2014 to May 2025 at the Affiliated Kunshan Hospital of Jiangsu University in Suzhou, China. A total of 1,076 hospitalized patients diagnosed with newly detected OC were included. The diagnosis of OC primarily relied on clinical symptoms such as abdominal masses, persistent pain, and gastrointestinal issues, while excluding other potential gynecological disorders during the evaluation process (19). Key diagnostic methods included transvaginal ultrasound, computed tomography (CT), and serum markers such as CA-125, which effectively assist clinicians in the preliminary assessment of the disease’s presence (20).

The confirmation of the diagnosis was accomplished through histopathological examination of clinical biopsy samples. Furthermore, the staging of OC differentiates between early-stage (localized) and advanced-stage (metastatic) disease, depending on a comprehensive analysis combining imaging tests and clinical evaluations (21). Identifying risk factors, including family history, age, and genetic mutations related to BRCA1/2, is particularly crucial for early screening in high-risk populations (22). This knowledge enables effective monitoring and interventions for individuals at high risk (23).

This study created specified exclusion criteria to improve the reliability and comprehensiveness of the research findings. Patients who satisfied any of the subsequent criteria were excluded: (1) missing TG data (n=172), (2) missing SUA data (n=139), (3) self-reported use of lipid-lowering medications (n=22), or (4) missing covariate data (n=19). The exclusion criteria were implemented to maintain data integrity.

**Figure 1:**
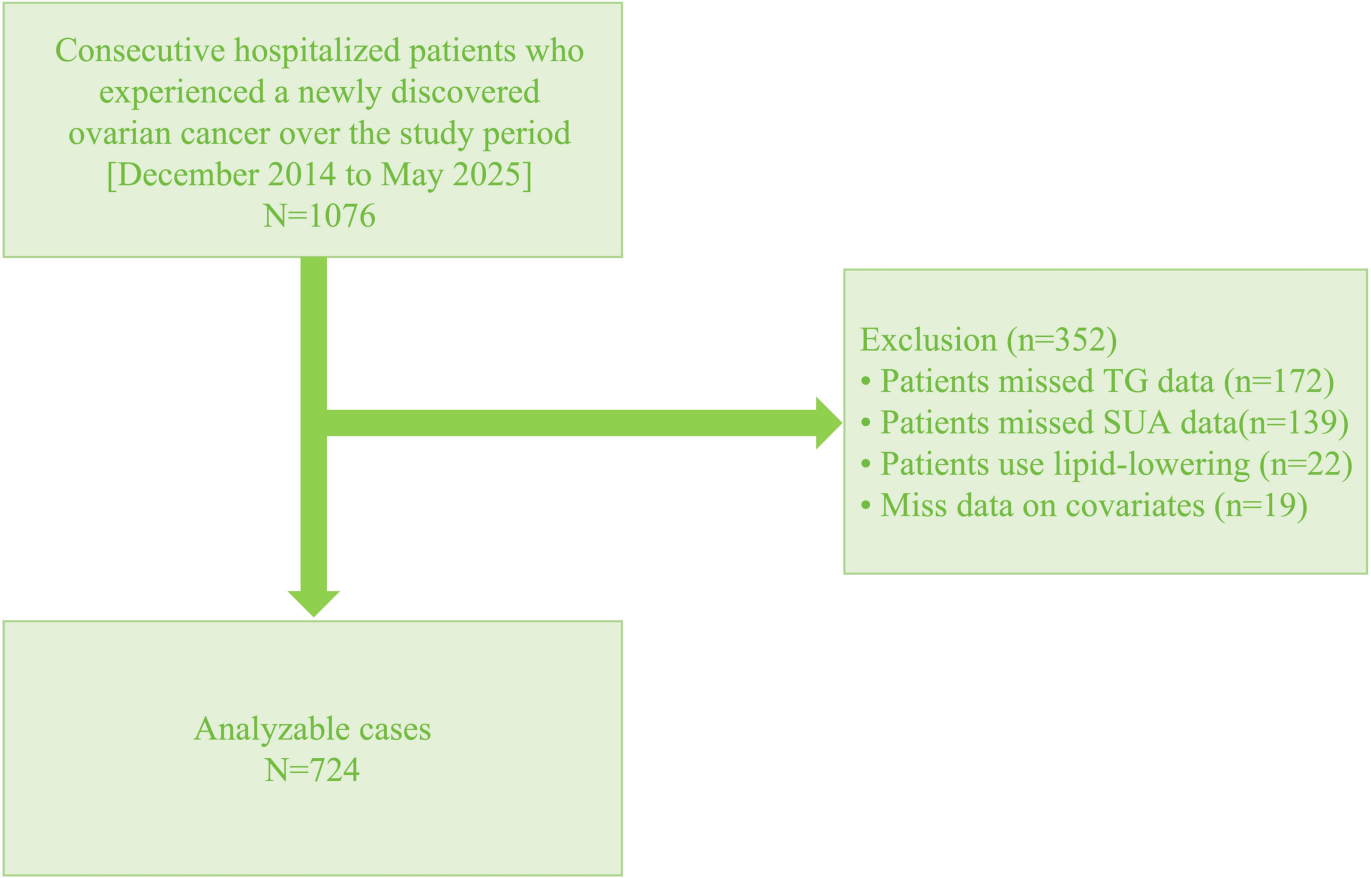
Study flow chart.

**Figure 1** presents an illustration that elucidates the patient selection process, enhancing readers’ comprehension of the exact inclusion and exclusion criteria. This visual aid serves not only to clarify the methodology but also to highlight the importance of adhering to these criteria for ensuring the validity of the study results. By providing a clear framework, it allows researchers to replicate the process in future investigations. Additionally, the study was approved by the Ethics Committee of the Affiliated Kunshan Hospital of Jiangsu University (Approval No. IEC-C-011-A04-V3.0). All participants provided signed informed consent for participation, data collection and publication, with assurances of anonymity and confidentiality.

### 2.2 Dependent variable and independent variable

In this study, TG levels were established as the independent variable, assessed through electrochemical luminescence immunoassay conducted on the Beckman AU5800 biochemical analyzer. SUA concentration was utilized as the dependent variable, assessed through an enzymatic colorimetric method. Clinical parameters were assessed by trained operators utilizing standardized instruments to guarantee data precision. Prior to daily examinations of participants, the analyzers underwent strict quality control procedures to maintain measurement reliability.

### 2.3 Covariates

Potential covariates considered in this analysis included age, body mass index (BMI), blood urea nitrogen (BUN), creatinine (Cr), high-density lipoprotein cholesterol (HDL-C), low-density lipoprotein cholesterol (LDL-C), and total cholesterol (TC) levels. All blood samples were collected in a fasting state to ensure consistency in the measurements. The usage of the following medications has also been included: Paclitaxel, Carboplatin, Bevacizumab, Hydrochlorothiazide, Furosemide, Aspirin, and Insulin. BUN and Cr reflect renal function, and since the kidney is the main route for SUA excretion, impaired renal function raises SUA. Adjusting for BUN and Cr reduces confounding of SUA–OC association by renal dysfunction. Dyslipidemia, via metabolic syndrome, inflammation, and insulin resistance, can affect both metabolic biomarkers and SUA handling. Adjusting for lipid profile variables helps control these pathways and better isolate OC-related association with SUA. Meanwhile, all included variables are based on existing literature support (17, 24–26).

### 2.4 Statistical analyses

This study presents continuous data as medians with interquartile ranges and categorical variables as counts and percentages. The examination of categorical data employed the Fisher’s exact test or the Pearson’s chi-squared test, whereas comparisons of continuous parameters utilized the t-test and the Mann-Whitney U tests. We employed linear regression models to investigate the association between TG levels and SUA concentrations in hospitalized patients with OC.

To further analyze the independent association between TG and SUA while accounting for potential covariates, multiple linear regression models were employed. Three models were developed: a minimally adjusted model and a fully adjusted model. The initial steps of the analysis included calculating the variance inflation factor (VIF) to identify collinearity among the covariates. The criteria for decision-making included: (1) a change of 10% or more in the regression coefficients (β) when adding a covariate to the unadjusted model or removing it from the fully adjusted model and (2) covariates that met the first criterion or exhibited a significance level of P < 0.01 in univariate analyses. The resulting models comprised Model 1 (unadjusted), Model 2 (adjusted for age and BMI), and Model 3 (additionally adjusted for BUN, Cr, HDL-C, LDL-C, TC, Paclitaxel, Carboplatin, Bevacizumab, Hydrochlorothiazide, Furosemide, Aspirin, and Insulin).

Alongside linear regression, general additive models (GAM) were utilized to examine the nonlinear association between TG and SUA concentrations (27). The threshold effects of the smoothed curves were identified using segmented regression, which is also called piecewise regression (28). Likelihood ratio tests (LRT) were employed to evaluate interactions and modifications among subgroups (29).

Statistical analyses were performed utilizing EmpowerStats (www.empowerstats.com, X&Y Solutions, Inc, MA, USA) and R version 3.6.3 (www.r-project.org) using linear regression models and generalized additive models. P < 0.05 was considered significant.

## 3 Results

### 3.1 Patient characteristics

**Table 1** presents the baseline characteristics of 724 OC patients admitted between December 2014 and May 2025, categorized according to TG levels. The average age of the patients was 56.94 ± 12.04 years, with a mean TG concentration of 1.73 ± 0.92 mmol/L and an average SUA level of 326.70 ± 102.57 μmol/L.

**Table 1:** Patient characteristics based on TG levels.

Characteristics	Mean+SD / N (%)
AGE (years)	56.94 ± 12.04
BMI (kg/m2)	23.06 ± 3.89
SUA (μmol/L)	326.70 ± 102.57
BUN (mmol/L)	5.85 ± 2.63
CR (umol/L)	77.81 ± 325.07
HDL (mmol/L)	1.36 ± 0.33
LDL (mmol/L)	2.95 ± 0.81
TC (mmol/L)	4.80 ± 1.05
TG (mmol/L)	1.73 ± 0.92
N (%)	
PACLITAXEL	
0	17 (2.35%)
1	707 (97.65%)
CARBOPLATIN	
0	17 (2.35%)
1	707 (97.65%)
BEVACIZUMAB	
0	618 (85.36%)
1	106 (14.64%)
HYDROCHLOROTHIAZIDE	
0	711 (98.20%)
1	13 (1.80%)
FUROSEMIDE	
0	665 (91.85%)
1	59 (8.15%)
ASPIRIN	
0	708 (97.79%)
1	16 (2.21%)
INSULIN	
0	673 (92.96%)
1	51 (7.04%)

TG, Triglycerides; SD, standard deviation; BMI, body mass index; SUA, Serum uric acid; BUN, Blood urea nitrogen; Cr, creatinine; HDL-C, High density lipoprotein-cholesterol; LDL-C, Low density lipoprotein cholesterol; TC, Total cholesterol.

### 3.2 Univariate analysis of factors linked with SUA

Univariate analyses demonstrated that SUA levels were significantly correlated with several clinical variables, including age, BMI, BUN, HDL-C, LDL-C, TC, Bevacizumab, Hydrochlorothiazide, Furosemide and Aspirin, as shown in **Table 2**. In contrast, no significant association were observed between SUA levels and the following variables investigated in this study: Cr, Paclitaxel, Carboplatin, and Insulin. This indicates that these factors may not substantially influence SUA levels in this population.

**Table 2:** Univariate analysis for SUA.

Characteristics	Mean+SD / N (%)	^a^β (95%CI) P-value
AGE (years)	56.94 ± 12.04	2.11 (1.50, 2.71) <0.01
BMI (kg/m2)	23.06 ± 3.89	5.72 (3.84, 7.59) <0.01
CR (umol/L)	77.81 ± 325.07	-0.01 (-0.03, 0.01) 0.40
BUN (mmol/L)	5.85 ± 2.63	14.84 (12.21, 17.47) <0.01
HDL (mmol/L)	1.36 ± 0.33	-48.25 (-70.32, -26.18) <0.01
LDL (mmol/L)	2.95 ± 0.81	15.20 (6.06, 24.34) 0.01
TC (mmol/L)	4.80 ± 1.05	8.73 (1.62, 15.84) 0.02
TG (mmol/L)	1.73 ± 0.92	41.07 (33.55, 48.60) <0.01
PACLITAXEL		
0	17 (2.35%)	0
1	707 (97.65%)	7.53 (-41.84, 56.90) 0.77
CARBOPLATIN		
0	17 (2.35%)	0
1	707 (97.65%)	7.53 (-41.84, 56.90) 0.77
BEVACIZUMAB		
0	618 (85.36%)	0
1	106 (14.64%)	-29.72 (-50.76, -8.68) 0.01
HYDROCHLOROTHIAZIDE		
0	711 (98.20%)	0
1	13 (1.80%)	90.93 (35.02, 146.84) 0.02
ASPIRIN		
0	708 (97.79%)	0
1	16 (2.21%)	52.58 (1.87, 103.30) 0.04
INSULIN		
0	673 (92.96%)	0
1	51 (7.04%)	-7.64 (-36.86, 21.57) 0.61
FUROSEMIDE		
0	665 (91.85%)	0
1	59 (8.15%)	199.99 (176.88, 223.10) <0.01

a The dependent variable was SUA and β is the result of univariate analysis for SUA.

SUA, Serum uric acid; SD, standard deviation; CI, confidence interval; BMI, body mass index; BUN, Blood urea nitrogen; Cr, creatinine; HDL-C, High density lipoprotein-cholesterol; LDL-C, Low density lipoprotein cholesterol; TC, Total cholesterol; TG, Triglycerides.

### 3.3 Investigation of the association between TG levels and SUA

Subsequently, we examined the association between TG and SUA levels in OC patients by developing three models (**Table 3**). Model 1, without adjustments, revealed a significant positive correlation (β = 41.07, 95% CI: 33.55 to 48.60, P < 0.01). The association continued significant in Model 2, adjusted for age and BMI (β = 35.76, 95% CI: 28.15 to 43.37, P < 0.01). Likewise, in Model 3, which had all relevant variables included (including age, BMI, BUN, Cr, HDL-C, LDL-C, TC, Paclitaxel, Carboplatin, Bevacizumab, Hydrochlorothiazide, Furosemide, Aspirin, and Insulin, the positive association continued to exist (β = 27.35, 95% CI: 19.82 to 34.87, P < 0.01).

**Table 3:** Association between TG concentrations and SUA level across various models (N=724).

Exposure	Model 1^a^ β (95%CI) P-value	Model 2^b^ β (95%CI) P-value	Model 3^c^ β (95%CI) P-value
TG Per 1mmol/ L increment	41.07 (33.55, 48.60) <0.01	35.76 (28.15, 43.37) <0.01	27.35 (19.82, 34.87) <0.01
TG quartile, N (%)			
Q1	Reference	Reference	Reference
Q2	52.46 (33.12, 71.81) <0.01	47.83 (28.91, 66.75) <0.01	35.36 (19.18, 51.53) <0.01
Q3	79.05 (59.78, 98.31) <0.01	70.72 (51.76, 89.68) <0.01	51.89 (35.58, 68.20) <0.01
Q4	117.10 (97.81, 136.40) <0.01	102.67 (83.18, 122.16) <0.01	90.08 (70.50, 109.66) <0.01
P-value for trend	<0.01	<0.01	<0.01

a No adjustment.

b Adjust for age, BMI.

c Adjust for age, BMI, BUN, CR, HDL, LDL, TC, Paclitaxel, Carboplatin, Bevacizumab, Hydrochlorothiazide, Furosemide, Aspirin, Insulin.

TG, Triglycerides; CI, confidence interval; Q1, first quartile; Q2, second quartile; Q3, third quartile; Q4, fourth quartile; BMI, body mass index; SUA, Serum uric acid; BUN, Blood urea nitrogen; Cr, creatinine; HDL-C, High density lipoprotein-cholesterol; LDL-C, Low density lipoprotein cholesterol; TC, Total cholesterol.

Grouping patients by TG quartiles revealed significantly elevated SUA levels in Q2, Q3, and Q4 relative to Q1. Model 3 indicated that the average SUA levels in Q2, Q3, and Q4 were incrementally higher by 8.01, 24.54, and 62.73 units, respectively, compared to Q1 (**Table 3**). This progressive elevation in SUA levels associated with ascending TG quartiles was consistently corroborated across all three models.

### 3.4 Spline smoothing plot and threshold analyses

**Figure 2:**
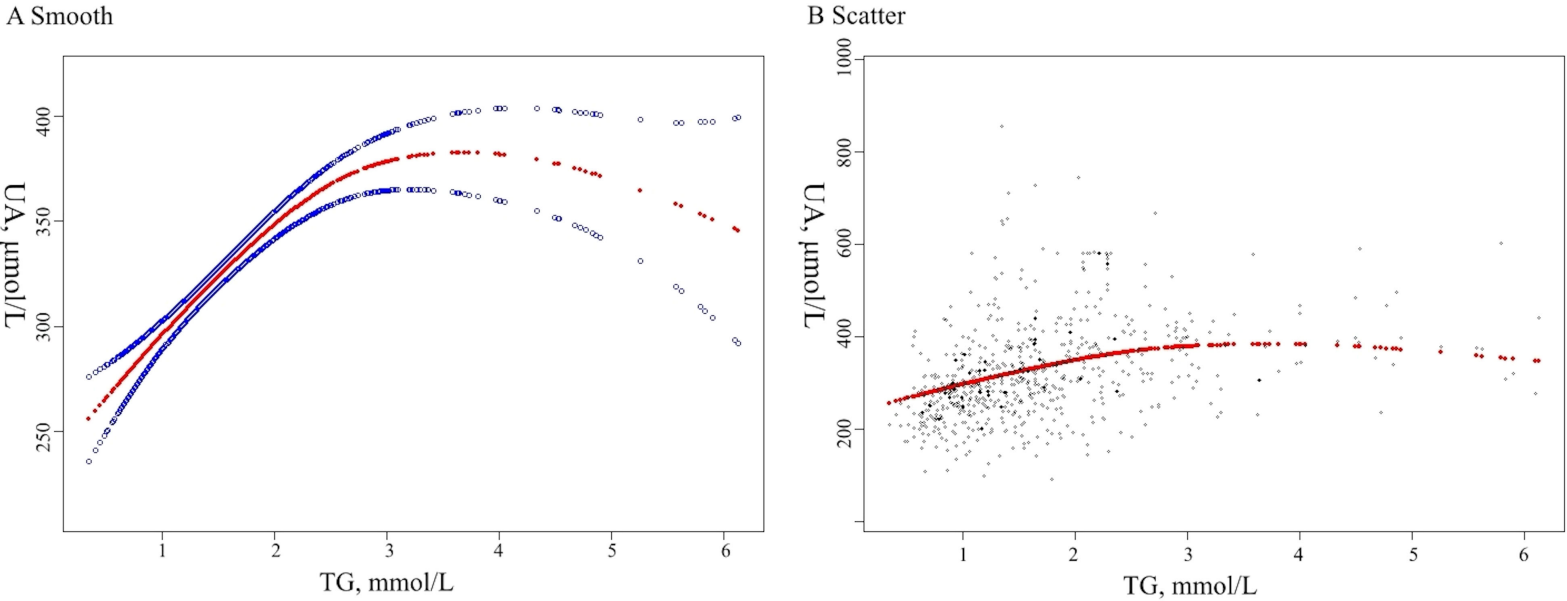
The curve fitting line for SUA and TG levels in OC patients **(A)** and scatter plot of the distribution for SUA and TG levels in OC patients **(B)** are shown. The model has been adjusted for variables including age, BMI, BUN, CR, LDL, HDL, TC, Paclitaxel, Carboplatin, Bevacizumab, Hydrochlorothiazide, Furosemide, Aspirin and Insulin. SUA, Serum uric acid; TG, Triglycerides; BMI, body mass index; BUN, Blood urea nitrogen; Cr, creatinine; LDL-C, Low density lipoprotein cholesterol; HDL-C, High density lipoprotein-cholesterol; TC, Total cholesterol.

To investigate the potential nonlinear association between TG and SUA levels, a GAM was employed. **Figure 2A** demonstrates a distinct nonlinear association following the adjustment for variables. including age, BMI, BUN, Cr, HDL-C, LDL-C, TC, Paclitaxel, Carboplatin, Bevacizumab, Hydrochlorothiazide, Furosemide, Aspirin, and Insulin. **Figure 2B** is a scatter plot illustrating the SUA and TG levels in patients with OC. To identify potential inflection points, a nonlinear correlation model was applied. This analysis revealed an inflection point for TG at 2.82 mmol/L. A notable positive correlation between SUA levels and TG was seen when TG levels were lower than 2.82 mmol/L (β =52.73, 95% CI: 41.99 to 63.74, P < 0.01). However, when TG levels exceeded 2.82 mmol/L, the association was not significant, with a β value of -14.39 (95% CI: -29.24 to 0.46, P = 0.06) (**Table 4**).

**Table 4:** Threshold effect analysis of the association between TG and SUA (N=724).

	Model3 ^a^ β(95%CI)	P-value
Model I ^b^		
One line effect	27.35 (19.82, 34.87)	<0.01
Model II ^c^		
TG turning point (K), mmol/L	2.82	
< K	52.73 (41.99, 63.47)	<0.01
> K	-14.39 (-29.24, 0.46)	0.06
Slope 2–Slope 1	-67.12 (-87.89, -46.34)	<0.01
LRT test ^d^	<0.01	

a Adjusted for age, BMI, BUN, CR, LDL, HDL, TC, Paclitaxel, Carboplatin, Bevacizumab, Hydrochlorothiazide, Furosemide, Aspirin, Insulin.

b Linear analysis, P value<0.05 indicates a linear relationship.

c Nonlinear analysis.

d LRT, likelihood ratio test, P-value <0.05 means Model II is significantly different from Model I, which indicates a nonlinear relationship.

TG, Triglycerides; CI, confidence interval; BMI, body mass index; SUA, Serum uric acid; BUN, Blood urea nitrogen; Cr, creatinine; HDL-C, High density lipoprotein-cholesterol; LDL-C, Low density lipoprotein cholesterol; TC, Total cholesterol.

The statistical analyses for this study were conducted using R packages provided by The R Foundation for Statistical Computing (http://www.R-project.org) and EmpowerStats, developed by X&Y Solutions, Inc, MA, USA (http://www.empowerstats.com).

## 4 Discussion

This cross-sectional analysis of 724 hospitalized OC patients reveals a compelling non-linear association between TG and SUA levels, with a critical inflection point at 2.82 mmol/L. Below this threshold, we noted a substantial positive association (β =52.73, 95% CI: 41.99 to 63.74, P < 0.01), while above this point, due to the small sample size and the wide confidence interval observed in patients with high TG levels, no significant association was established between the two variables.

However, evidence from Mendelian randomization studies provides no clear evidence for a causal role of SUA in increasing TG levels (30). Nevertheless, most previous studies have identified a significant positive correlation between the two variables. Additionally, several studies have reported similar thresholds, below which a significant positive correlation exists, while above this threshold, the two variables show no correlation (17, 24–26).

The association between elevated TG and increased SUA is likely underpinned by multiple interconnected pathways that reflect metabolic dysregulation. Fatty acid synthesis and TG formation require ATP as their energy source; therefore, a depletion of ATP can lead to an accumulation of AMP, which subsequently promotes excessive production of SUA (31). Additionally, high levels of TG may contribute to oxidative stress through the increased generation of reactive oxygen species during lipid metabolism (32). This oxidative stress, in turn, can enhance the activity of xanthine oxidase, an enzyme that plays a critical role in SUA production (33). Furthermore, it could be hypothesized that insulin resistance, which is often observed in OC patients, may simultaneously promote TG synthesis while impairing SUA excretion through enhanced renal tubular reabsorption. This condition might lead to a scenario where elevated TG levels coexist with increased SUA, potentially exacerbating metabolic dysfunction and contributing to the overall disease state (34).

The threshold effect observed at 2.82 mmol/L may indicate a potential metabolic tipping point where compensatory mechanisms might be overwhelmed, potentially contributing to metabolic dysfunction commonly correlated with advanced cancer states. Therefore, it is possible that once SUA levels exceed a certain threshold, the positive link between TG and SUA may weaken and become statistically insignificant. However, this remains a hypothesis that requires further investigation.

Our findings significantly advance the existing literature by providing the first quantitative threshold for TG-SUA interaction in OC patients. For instance, elevated SUA levels in OC patients have been reported, but the association with lipid metabolism remains underexplored (35). Similarly, metabolic syndrome studies in cancer populations have identified association between TG and SUA but lacked the precision of threshold identification (36). The novelty of our study is reflected in several aspects. Firstly, we identified a possible TG threshold that may have clinical relevance; secondly, we demonstrated a non-linear association between TG and SUA, rather than a conventional linear association; and finally, we integrated comprehensive metabolic profiling in a cross-sectional study of OC.

This study has several important limitations that should be acknowledged. First, the results derived from this single-center cross-sectional study conducted exclusively in a Chinese population may have limited generalizability to other ethnic groups and healthcare settings. The homogeneous patient population from one geographic region restricts our ability to account for genetic polymorphisms, cultural dietary patterns, varying treatment protocols, and healthcare system differences that could significantly influence the TG-SUA correlation. The observed threshold of 2.82 mmol/L may not be universally applicable across different ethnic populations due to potential variations in lipid metabolism, purine metabolism pathways, and baseline metabolic profiles. Additionally, there were insufficient adjustments for critical variables such as cancer staging and dietary factors, which further restricts the applicability of our results. Moreover, we excluded patients on lipid-lowering therapy to avoid medication effects on blood lipids and related biomarkers, allowing for clearer assessment of disease-related association. However, this exclusion may limit the generalizability of our findings to those receiving such therapy; thus, applicability to that group should be interpreted with caution. We recommend future studies include larger populations with patients on lipid-lowering medications to evaluate any pharmacologic impact.

Furthermore, the low number of patients with elevated SUA levels and the wide confidence intervals for those above the threshold indicate significant uncertainty in our estimates. These intervals encompass both positive and negative values, reflecting variability in the data. Future research should include multi-center studies with diverse populations to enhance generalizability and longitudinal designs to establish temporal association between TG and SUA levels. Moreover, the retrospective cross-sectional design fundamentally precludes causal inference and may introduce selection bias. This design limitation means our findings represent associations rather than causal relationship. The observed correlations could be explained by unmeasured confounding factors, reverse causation, or common underlying pathways affecting both TG and SUA levels simultaneously. Our article also lacks indicators such as cancer staging and histologic subtype, which would provide more depth to the analysis. Comprehensive collection of data on dietary habits, treatment regimens, cancer staging, and histological subtypes is crucial for understanding their role in SUA metabolism, which will provide important insights for identifying risk factors for hyperuricemia, early prevention, and personalized treatment. Additionally, exploring genetic and environmental influences will also help identify related risk factors.

AHA/ACC, NCEP ATP III, ESC/EAS and most national guidelines, including those from China, generally consider TG lower than 1.7 mmol/L as normal, 1.7–2.2 mmol/L as borderline/mildly elevated, 2.3–5.6 mmol/L as moderately elevated, and greater than or equal to 5.6 mmol/L as severely elevated. The TG value of 2.82 mmol/L therefore falls within the elevated range. According to current guidelines, maintaining TG below guideline thresholds may help limit SUA production; moreover, keeping TG within the normal range could be beneficial for preventing hyperuricemia and protecting renal function. In our study, the extremely high TG subgroup was small and exhibited large variance, producing unstable estimates; this may explain the loss of statistical significance observed above the 2.82 mmol/L threshold. Validation in larger, multiethnic cross-sectional is required to confirm the robustness of this finding. The identification of the 2.82 mmol/L threshold offers clinicians a specific parameter for risk stratification and intervention planning. Patients with TG levels below this threshold may benefit from intensive metabolic monitoring, as elevated SUA within this range indicates active metabolic dysregulation that could influence cancer progression and treatment response. By recognizing this threshold, healthcare providers can tailor their approaches to ensure at-risk patients receive appropriate attention. Lifestyle modifications to reduce TG levels through dietary changes and exercise may help maintain SUA within optimal ranges, potentially reducing inflammation and oxidative stress that contribute to tumor progression. Additionally, routinely monitoring both TG and SUA could serve as early indicators of metabolic decompensation, allowing for timely intervention before complications arise, particularly in those at risk of metabolic syndrome. Given the cross-sectional nature of this study, future research should prioritize prospective longitudinal designs to establish temporal association and potential causality between TG and SUA levels. Randomized controlled trials investigating TG-lowering interventions and their effects on SUA levels would provide stronger evidence for clinical recommendations.

Future research should focus on several key aspects. Firstly, since this study is cross-sectional, it can only establish a potential connection between SUA and TG. Further laboratory research is necessary to elucidate the specific mechanisms underlying this association. Additionally, future studies should incorporate a broader range of variables, such as cancer stage and tissue subtype, when conditions permit. It is also essential to conduct experiments across different regions and among diverse populations to validate the findings’ generalizability. Specifically, large-scale international collaborative studies encompassing diverse ethnic populations (Caucasian, African American, Hispanic, East Asian, South Asian, and Middle Eastern populations) are critically needed to validate our findings. These studies should aim to establish whether the identified TG threshold of 2.82 mmol/L demonstrates universal clinical relevance or requires population-specific adjustments across different genetic backgrounds and healthcare environments.

## 5 Conclusions

In summary, this analysis of 724 hospitalized OC patients revealed a significant association between TG and SUA levels, with this association being particularly significant in the TG values lower than 2.82 mmol/L. These associative findings suggest that TG levels may potentially serve as a biomarker for SUA-related metabolic disturbances among OC patients. However, prospective longitudinal studies are needed to establish causality and determine whether TG-targeted interventions could influence SUA levels in clinical practice. Further research using larger, multi-ethnic cross-sectional studies is warranted to validate these findings.

## Data Availability

The original contributions presented in the study are included in the article/supplementary material. Further inquiries can be directed to the corresponding author/s.
